# Bilateral Temporal Headache As the Presenting Symptoms for a Case of Graves’ Disease

**DOI:** 10.7759/cureus.51344

**Published:** 2023-12-30

**Authors:** Ahmad S Alsabban, Hisham M Bakri, Abeer Z Abduljabbar, Abdullah A Almesfer, Abdulkareem M Alturkistani

**Affiliations:** 1 Family Medicine, Ministry of the National Guard-Health Affairs, King Abdulaziz Medical City, Jeddah, SAU; 2 Family Medicine, College of Medicine, King Saud Bin Abdulaziz University for Health Sciences, Jeddah, SAU; 3 Family Medicine, King Abdullah International Medical Research Center, Jeddah, SAU; 4 General Medicine, King Abdulaziz University Faculty of Medicine, Jeddah, SAU; 5 Medicine, King Abdulaziz University Faculty of Medicine, Jeddah, SAU

**Keywords:** thyroid related disease, unilateral exophthalmos, graves' disease, hyperthyroidism, headache

## Abstract

Headache is not typically a presenting symptom of Graves' disease. Only a few studies suggest hyperthyroidism can cause headaches, and the connection remains indeterminable. While some cases report hyperthyroidism presenting as a simple headache, it is not specific to Graves' disease. This report details a middle-aged male patient who visited a primary healthcare center with a two-month history of bilateral temporal headaches. He was initially diagnosed as a query case of temporal arteritis due to his age and affected area and was transferred to the emergency department for a comprehensive evaluation. After discussions with specialists and various laboratory tests and CT scans, he was diagnosed with Graves' disease. Treatment led to fast improvement and relief of his headaches. This case represents a rare instance of Graves' disease where the patient's sole complaint was a simple headache without any other symptoms. We advise doctors to consider diseases related to increased thyroid gland activity when dealing with headaches.

## Introduction

Headache is a very common complaint as 96% of people experience it at least once in their lifetime [1]. Moreover, in a systematic review done in 2018, headache ranked as the sixth highest patient-reported reason for visiting a primary health center [2]. Although primary headache disorders form the majority of the differential diagnoses, secondary headache disorders remain an important cause of headache which clinicians must keep in mind. This entity is well studied and is categorized into eight different subtypes in the International Classification of Headache Disorders (ICHD) [3]. Hyperthyroidism is not listed amongst the most common causes of secondary headache in a study that included multiple centers located in Asia, Africa, The Middle East, and Turkey [4]. On the other hand, hyperthyroidism disorders like Graves' disease are also another highly prevalent entity; with the prevalence of suspected cases in Jeddah, Saudi Arabia, where the case to be reported is from, being 1.2% [5]. A 10-year study done in a hospital in Saudi Arabia in 2008 did not include headache among the 25 top presentations of Grave’s disease [6]. In addition to the lack of a reported relationship between the two entities, we reviewed the literature and found only two cases where headache was the presenting complaint of subacute thyroiditis, and painless thyroiditis, respectively, and none where headache was the presenting complaint of Graves' disease [7,8].

## Case presentation

On July 20, 2017, a 55-year-old male, with no significant past medical history, presented to a primary health care center with complaints of intermittent headaches in the bilateral temporal area for the past two months. The severity had increased over the last two weeks, with each episode lasting 30 minutes. The patient did not take any analgesics, but the pain was relieved by rest and sitting in a dark room. The headaches were associated with blurred vision, left eye redness, pain, and lacrimation. There was no history of dizziness or B symptoms (decreased weight or appetite, weight loss, fever, and night sweats) and the remainder of the systemic review was unremarkable. Upon examination, the patient appeared generally well, not in distress, with vitals within the normal range. Central nervous system examination revealed a Glasgow Coma Scale score of 15/15, preserved motor function, normal power, tone, and reflexes. Visual acuity was scored 6 out of 6, with tenderness over the right temporal area. Due to the patient's age and the area affected, the family physician referred the patient to the Emergency Department as a case of query temporal arteritis. In the ER, the patient's condition remained the same. A standard blood test (complete blood count, basic metabolic panel and comprehensive metabolic panel) and a brain CT were performed. Internal Medicine, Rheumatology and Neurology departments were consulted. The brain CT showed no acute brain insult, and all the requested labs were within normal range. The patient was given an outpatient appointment with Neurology. Moreover, Internal Medicine referred the patient to the Ophthalmology Department for suspected anterior ischemic optic neuritis. Furthermore, the Neurology Department was consulted, and their impression was a diagnosis of migraine headache. After that the Internal Medicine team saw the patient in the ER, revealing right eyelid retraction, which was subtle and not picked up by other teams. Management consisted of eye lubricants for the right eye and the patient was discharged home.

On July 23, 2017, the family medicine physician reviewed the case again. The thyroid panel results were available and indicated hyperthyroidism with a thyroid stimulating hormone (TSH) level of <0.01 MIU/L (normal range: 0.4 - 4.0 MIU/L) and free T4 level of 28.2 μg/dL (normal range: 5.0 - 12.0 μg/dL) (Table 1). Accordingly, the patient was referred to the Endocrinology Department.

**Table 1:** Laboratory test results WBC: White blood cells, RBC: Red blood cells, HGB: Hemoglobin, HCT: Hematocrit, MCV: Mean corpuscular volume, MCHC: Mean corpuscular hemoglobin concentration, RDW: Red cell distribution width, ESR: Erythrocyte sedimentation rate, Eos auto%: Eosinophil count, neutro Auto%: Neutrophil count in percentage, TSH: Thyroid stimulating hormone, T4: Thyroxine, BUN: Blood urea nitrogen, CO2: Carbon dioxide, AGAP: Anion gap, AST: Aspartate aminotransferase, Alk phos: Alkaline Phosphatase

Reg. Date	Exam.	Result	Reference Range
20-Jul-17 11:56	WBC	6.1× 10^9^/L	(4.5 - 11.0× 10^9^/L)
20-Jul-17 11:56	RBC	6.6 mcL	4.0 - 5.9 mcL
20-Jul-17 11:56	HGB	16.5 g/dL	14.0 - 18.0 g/dL
20-Jul-17 11:56	HCT	52.4%	41.0 - 50.0%
20-Jul-17 11:56	MCV	79.3 fl	80.0 - 100.0 fl
20-Jul-17 11:56	MCH	24.9 pg/cell	27.0 - 33.0 pg/cell
20-Jul-17 11:56	MCHC	31.4 g/dL	33.0 - 36.0 g/dL
20-Jul-17 11:56	RDW	13.2%	12.0 - 15.0%
20-Jul-17 11:56	ESR	17 mm/hr	<15.0 mm/hr
20-Jul-17 11:56	Eos Auto%	2.00	0.0 - 6.0
20-Jul-17 11:56	Neutro Auto%	47.10	55.0 - 70.0
20-Jul-17 18:54	TSH	<0.01MIU/L	0.4 - 4.0 MIU/L
20-Jul-17 18:54	Free T4	28.2 μg/dL	5.0 - 12.0 μg/dL
16-Oct-17 10:45	Creatinine	61 μg/dL	61.9 - 114.9 μg/dL
16-Oct-17 10:45	Total Protein	76 g/dL	60.0 - 83.0 g/dL
16-Oct-17 10:45	BUN	3.5 mmol/L	2.1 - 8.5 mmol/L
16-Oct-17 10:45	CO2	25 mmol/L	23.0 - 29.0 mmol/L
16-Oct-17 10:45	Chloride	105 MEq/L	96.0 - 106.0 MEq/L
16-Oct-17 10:45	Potassium	5.3 mmol/L	3.6 - 5.2 mmol/L
16-Oct-17 10:45	Sodium	137 mEq/L	135 - 145 mEq/L
16-Oct-17 10:45	AGAP	7 mEq/L	4.0 - 12.0 mEq/L
16-Oct-17 10:45	Globulin	38 g/dL	20.0 - 35.0 g/dL
16-Oct-17 10:45	AST	20 units/L	8.0 - 33.0 units/L
16-Oct-17 10:45	Albumin	41 g/dL	35.0 - 55.0 g/dL
16-Oct-17 10:45	Alk Phos	188 IU/L	44.0 - 147.0 IU/L
16-Oct-17 10:45	Total bilirubin	13.6 mg/dL	1.71 - 20.5 mg/dL

**Figure 1:**
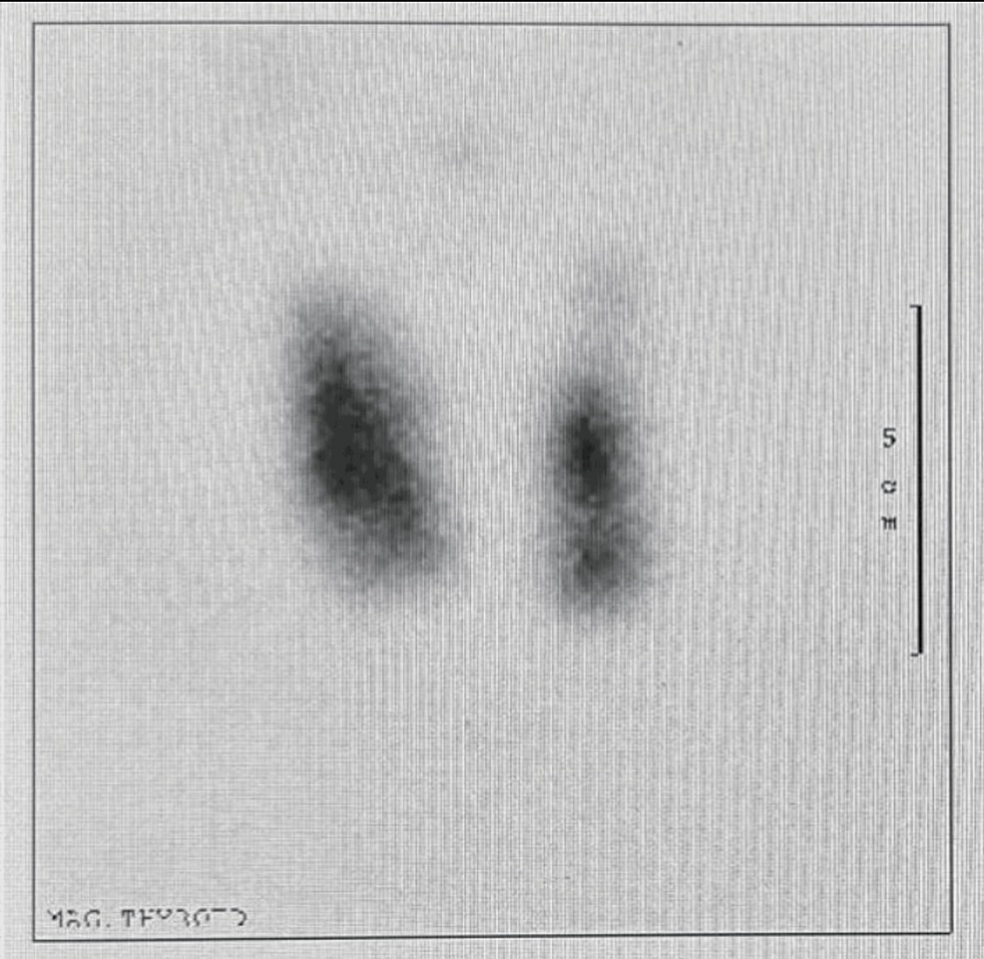
Thyroid scan and uptake Findings: Scan shows hyperemia in thyroid region. On uptake images there is fairly uniform tracer uptake throughout the gland. Thyroid uptake 6.5% normal 1-4%. Impression: The scan findings are highly suggestive of Graves' disease.

On October 16, 2017, the patient attended the Endocrinology OPD appointment, exhibiting a new swelling of the right eye, with lid lag and retraction. He complained of pain with movement, and the conjunctiva was erythematous. A fine action tremor was noted, but no other signs, symptoms, or examination findings of hyperthyroidism were present. The impression was a query for Graves' ophthalmopathy. Then, a thyroid uptake scan was ordered, and the thyroid panel was repeated. The patient was discharged on 10 mg of propranolol twice daily as symptomatic treatment for tachycardia, with a follow-up appointment after one week. A week later, the thyroid iodine uptake scan showed diffuse, uniform iodine uptake at 6.5% (normal <1%), indicative of Graves' disease (Figure 1). The patient was prescribed 5 milligrams of methimazole once daily, with an OPD follow-up appointment after one month. In the follow-up appointment, the patient stated that the headache had subsided completely since the initiation of the anti-thyroid medication. He continued to follow up with Endocrinology for further management of Graves’ disease.

## Discussion

Graves’ disease stands out as the leading cause of hyperthyroidism, characterized by an autoimmune condition associated with circulating TSH receptor autoantibodies. These antibodies lead to excessive stimulation of the thyroid gland, resulting in an overproduction of thyroid hormones. Notably, thyroid-related diseases are typically not considered in current approaches to isolated headaches in adults, as reports on the association between headaches and Graves’ disease are scarce. Furthermore, a thyroid panel is usually reserved for patients presenting with the most commonly reported hyperthyroid symptoms, including heat intolerance (86%), tiredness (53%), weight loss (53%), exophthalmos (50%), palpitations (49%), excessive sweating (48%), increased appetite (45%), dyspnea on effort (36%), sweaty palms (36%), nervousness (35%), oligomenorrhea (14.7%), menorrhagia (5%), and infertility (4.7%) [9].

According to our literature, this case represents the first detailed report on the occurrence of a headache as the presenting complaint of Graves’ disease. The case underscores the rare presentation of the relationship between headache disorder and Graves’ disease. In this instance, a middle-aged male experienced intermittent bilateral temporal pain headaches with associated symptoms such as blurred vision, left eye redness, pain, and lacrimation. Despite the initial diagnosis being temporal arteritis based on age and presenting symptoms, arriving at the correct diagnosis posed a challenge. Discussions and evaluations with multiple medical teams, including the neurology team, led to the opinion that the most likely differential diagnosis was migraine.

Notably, the subtle right eyelid retraction observed by the ophthalmology team became a crucial clue in the diagnostic process. Ordering thyroid function tests and referring the patient to endocrinology based on the results were essential steps in taking further action in the management process. This case highlights the complexity of diagnosing isolated symptoms like headaches, necessitating collaboration across multiple medical teams to explore underlying systemic disorders such as hyperthyroidism.

On the other hand, we reviewed two other similar reported cases discussing the relationship between headaches and subacute thyroiditis, as well as painless thyroiditis, both involving male patients. The first case involved a middle-aged man, aged 52, who initially complained of headaches. His doctors misdiagnosed him with meningitis based on laboratory and radiological findings. However, his symptoms did not improve with meningitis treatment. Later on, they discovered a thyroid abnormality through additional tests, including a thyroid panel, leading to the diagnosis of subacute thyroiditis. He was then treated based on this diagnosis, and his symptoms improved [7]. In contrast to our patient and the first case, the second case involves an 18-year-old male who was complaining of headaches. Based on his history and laboratory findings, he was diagnosed with painless thyroiditis. Following the management, the headache improved [8].

The pathophysiological mechanism explaining how hyperthyroidism could lead to headaches remains unclear. In the previously mentioned study, there was a discussion about increased intracranial pressure resulting from hyperthyroidism, leading to elevated cerebral blood flow. Consequently, the patient reported experiencing headaches. Another suggested possibility is that hyperthyroidism may cause vasospasm, leading to headaches. Moreover, a theoretical framework suggests that thyroid hormones could trigger migraines by promoting oxidative stress in the brain and enhancing cortical excitability through the reduction of gamma-aminobutyric acid synthesis [7]. Some studies indicate that thyroid issues are common in chronic daily headaches, so screening may be needed [10,11]. However, a different study found that ordering thyroid function tests has only a small impact on managing headaches [12]. Another study contradicts this idea [13]. In our case, requesting a thyroid analysis test proved highly beneficial and played a crucial role in reaching a diagnosis. We suggest that more studies are needed to determine the relationship between thyroid hormone abnormalities and headaches and whether thyroid testing is necessary for individuals with new or persistent headaches.

## Conclusions

This case represents a rare instance of Graves' disease where the patient's sole complaint was a simple headache and subtle eyelid retraction with no other symptom. Due to this information, alongside the benefits of early diagnosis of Graves' disease, we recommend that thyroid-related disease be considered more when approaching a patient with recurring headaches with no apparent cause. This could be in the form of a focused thyroid examination or investigation in patients presenting with non-typical headaches.
